# Educational intervention on water intake improves hydration status and enhances exercise performance in athletic youth

**DOI:** 10.1111/j.1600-0838.2011.01296.x

**Published:** 2012-10

**Authors:** S A Kavouras, G Arnaoutis, M Makrillos, C Garagouni, E Nikolaou, O Chira, E Ellinikaki, L S Sidossis

**Affiliations:** Laboratory of Nutrition & Clinical Dietetics, Department of Nutrition and Dietetics, Harokopio UniversityAthens, Greece

**Keywords:** urine specific gravity, fluid ingestion, sport camp, hydration state, dehydration, children, environmental symptoms questionnaire

## Abstract

We aimed to evaluate whether an intervention program emphasizing in increased fluid intake can improve exercise performance in children exercising in the heat. Ninety-two young athletes participated in the study (age: 13.8 ± 0.4 years, weight: 54.9 ± 1.5 kg). Thirty-one (boys: 13, girls: 18) children served as the control group (CON) and 61 (boys: 30, girls: 31) as the intervention (INT). Volunteers had free access to fluids. Hydration was assessed on the basis of first morning urine. A series of field tests were used to evaluate exercise performance. All tests occurred outdoors in the morning (mean ambient temperature=28 °C). After baseline testing, INT attended a lecture on hydration, and urine color charts were mounted in all bathrooms. Additionally, water accessibility was facilitated in training, dining and resting areas. Hydration status was improved significantly in the INT [USG: pre=1.031 ± 0.09, post=1.023 ± 0.012, *P*<0.05; urine osmolality (mOsm/kg water): pre=941 ± 30, post=782 ± 34, *P*<0.05], while no statistically significant changes were found in the CON [USG: pre=1.033 ± 0.011, post=1.032 ± 0.013, *P*>0.05; urine osmolality (mOsm/kg water) 970 ± 38 vs 961 ± 38, *P*>0.05]. Performance in an endurance run was improved significantly only in INT (time for 600 m: pre=189 ± 5 s, post=167 ± 4 s, *P*<0.05). Improving hydration status by ad libitum consumption of water can enhance performance in young children exercising in the heat.

Water and electrolyte balance are critical for the function of all organs and indeed, for maintaining health in general. It has been shown that losses as small as 2% of body weight (BW) increase significantly physiologic strain (Gonzalez-Alonso et al., 1995), decrease exercise performance (Montain & Coyle, 1992; Gonzalez-Alonso et al., 1997) and hinder the thermoregulatory advantages conferred by high aerobic fitness (Cadarette et al., 1984) and heat acclimatization (Sawka et al., 1983).

It has been also documented that even when water or sports drink availability is present, inadequate fluid intake during exercise under warm conditions is disadvantageous for both children and adults (Bar-Or et al., 1980; Meyer et al., 1995). Early studies have indicated that children usually exhibit lower maximal aerobic capacity, higher adiposity and less sweat than do adults when exercising in warm environments, due to their greater body surface area-to-mass ratio, which in turn leads to faster heat absorption from the environment when ambient temperature exceeds skin temperature (Epstein et al., 1983; Falk et al., 1992a, b; Marino et al., 2000). This fact limits their reliance in evaporative heat loss (Meyer et al., 1992). Furthermore, children, similar to adults, do not drink enough to adequately replace water losses under warm conditions and exhibit hypohydration even when fluids are provided ad libitum, a phenomenon described as involuntary dehydration (Bar-Or et al., 1980; Greenleaf, 1992; Wilk & Bar-Or, 1996). However, contrary to the aforementioned observations, current research information fails to indicate thermoregulatory differences in the heat between children and adults, demonstrating that data in this specific field are not entirely clear (Falk & Dotan, 2008; Rowland, 2008).

Furthermore, little information is available concerning the capacity of children exercising in the heat. The majority of the research to date has been performed in sedentary non-acclimatized children under controlled environments (Bar-Or et al., 1980; Meyer et al., 1995; Wilk & Bar-Or, 1996; Iuliano et al., 1998). Additionally, there is a lack of studies investigating the relationship between the hydration status of children and their physical performance in free-living situations. To our knowledge, the relation of hydration status in children exercising in free-living situations has not been investigated systematically (Wilk et al., 2007; Rivera-Brown et al., 2008). A recent work by McDermott et al. (2009) showed that children arrived at a football camp hypohydrated and maintained this condition throughout their stay leading to impaired recovery and subsequent performance. The educational intervention used did not reveal a clear change in hydration status.

It was hypothesized that facilitation of water intake will lead to significant enhancement in the field tests. Therefore, the purpose of the present study was to investigate the effect of a nutrition intervention program emphasizing water consumption on the prevention of dehydration, and in addition, study the effects of hydration improvement in the physical performance of young athletes.

## Materials and methods

Data collection occurred in a summer training camp during two consecutive 5 days in Loutraki, Greece during August 2007. A total of 92 young trained volleyball or basketball athletes participated in the study; their physiological characteristics are presented in Table 1. In the first week, 31 children (boys: 13, girls: 18) served as the control group (CON) and at the second week 61 children (boys: 30, girls: 31) served as the intervention group (INT). Although this study design, where two completely separate sets of youths were used, may have had some limitations concerning the between-week differences in environmental factors however, this was unavoidable due to all the extra hydration info provided in the INT group. The study was approved by the University human subjects committee and all volunteers and their parents were informed about the nature of the study and gave informed written consent. All subjected were healthy, active individuals and trained regularly at least for 2 years (at mean: three times per week) and had a normal BMI for age, according to Cole criteria (Cole et al., 2007). A medical history questionnaire was used to exclude participant with conditions that could affect the interpretation of our data, including:

**Table 1:** Physiological characteristics of the study participants

Characteristics	*n*=92	CON=31	INT=61
Age (years)	13.8 ± 4.2	13.2 ± 2.3	14.0 ± 4.8
Gender (men/women)	43/49	13/18	30/31
Weight (kg)	54.9 ± 13.7	55.75 ± 2.5	54.5 ± 13.7
Height (cm)	164 ± 13.9	165 ± 14.8	163.1 ± 13.4
Body fat (%)	19.2 ± 7.3	18.8 ± 7.4	19.4 ± 7.2

Values are means ± SE.

- Evidence of clinically relevant cardiovascular, hematologic, hepatic, gastrointestinal, renal, pulmonary, endocrine or psychiatric history of disease.
- Surgical operation on digestive tract, except possible appendectomy.
- Regular drug treatment within 15 days before start of the study.
- Inability to participate in the entire study.

### Protocol

The daily program in the camp consisted of an early morning workout, a mid-day rest and an evening training. Hydration status was assessed in the morning of the second (pre) and fourth (post) day of the camp (08:00 hours) based on the first morning urine sample and BW was also measured. After a standardized breakfast-snack, a specific warm-up was performed, including mainly light running and basic stretching exercises. Afterward, children were divided into groups and performed a series of field performance tests randomly: 600 m maximum running test, 30 m maximum sprint running test, vertical jump and a skill test for volley ball and basketball. Vertical jump was measured by getting the children to reach up against a flat wall, with a flat surface under their feet and mark off the highest point they could reach flat-footed (standing height). Then, they were instructed to complete three jumps from a squatting position, marking off the highest point they could reach. The distance between the two marks (standing height vs highest point) was measured and served as their vertical jump. The skill test in volleyball was the number of successful service out of five trials that will reach a specific metal rectangular target (130 × 78 × 68 cm) on the opposite part of the court. For basketball, the skill test consisted of the number of point scored in five consecutive free throws from the keyhole. Heart rate was measured during the tests with telemetry and it was recorded immediately after the end of the 600 m and 30 m run. The performance tests were performed only in the second and fourth day of the children's stay in the camp, without any earlier familiarization, in order to avoid any biased results due to learning effect. The environmental temperatures for days 2 and 4 for the control group were 27.0 and 29.0 °C and wet bulb temperature (WBGT) 21.5 and 20.0 °C, respectively, while for the intervention group environmental temperatures were 29.0 °C for day 2 and 28 °C for day 4, while WBGT was 20.0 and 21 °C, respectively. Statistical comparisons between WBGT values for both groups did not reveal any significant differences.

Bottled water (Volvic®, Puy De Dome, France) was distributed to all camp facilities, while kids had free access to the camp's cafeteria, which also provided sport and soft drinks. Nevertheless, according to a specific Food Frequency Questionnaire that was provided, most of the children were drinking mainly water.

### Hydration assessment

Hydration status was assessed from first morning urine samples based on urine specific gravity (USG) and urine osmolality (Uosm). Cut off point for euhydration was based on USG<1.020 and Uosm<700 mOsm/kg water, according to the ACSM position statement (Sawka et al., 2007). USG was measured with a table-based refractometer (Gast Manufacturing Inc., Benton Harbor, Michigan, USA). Urine osmolality was measured in duplicate, by freezing point depression (3D3 Advanced Osmometer, Advanced Instruments Inc., Norwood, Massachusetts, USA). A modified version of the environmental symptom questionnaire (Sampson & Kobrick, 1980) was used at the end of the performance tests both pre- and post-intervention. An exit camp questionnaire was also provided on day 5 in order to assess volunteers perception regarding their fluid intake and practical issues related to fluid access.

### Intervention plan

Following the second day evaluation in the control group, no instructions or feedback was given to the kids, but they had free access to fluids as they do during typical workouts or games. In the intervention group following the hydration assessment and the performance testing, the athletes participated in an intervention program that consisted of the following:

- One-hour lecture on hydration and its benefits, during which subjects were given instructions for maintaining optimal hydration. Verbal and written key points for maintenance of good hydration status were also provided.
- The urine color chart (Armstrong et al., 1994, 1998) was explained and color copies were mounted in all bathrooms of the camp facilities.
- Improved water accessibility throughout the facilities of the sports camp, in training, dining and rest areas with bottles of water placed everywhere.
- During some of the workouts, athletes were weighted before and after with minimal clothing to identify fluid losses during the workouts, while no instructions were given.

The control group had exactly the same conditions except that no educational intervention was provided.

Authorized persons performed data entry in a blind fashion using a personal computer equipped with statistical software SPSS 16. For purposes of data control, a second, authorized person re-entered the data; and then, a computerized cross-control was performed to assess data conformity. Once data conformity was achieved, the data were considered clean and ready for analysis. Data are presented as mean ± SE or frequencies. A two-way analysis of variance model was used to check differences between the two groups and over time (pre- and post-intervention) in normally distributed data. Normality was tested by the Shapiro–Wilk test. For non-normally distributed variables, their log-transformation was used to test differences. Differences in demographic characteristics as well as changes between groups were tested by one-way analysis of variance. A type I error of 0.05 or less was the threshold for statistical significance.

## Results

**Fig. 1:**
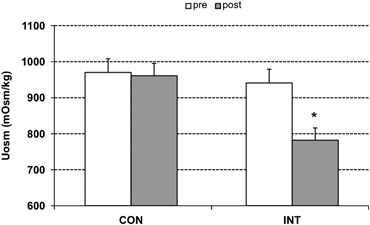
Urine specific gravity (USG) before and after the intervention in the control and intervention group. ^*^Statistically significant difference between pre and post in each group.

The 92 young trained volleyball and basketball athletes participated in the study, were separated in two groups, the control (CON) (age 13.3 ± 0.4 years, weight 55.7 ± 2.5 kg, height 1.66 ± 0.25 m and body fat 18.9 ± 1.5%) and the intervention (INT) group (age 14.1 ± 0.6 years, weight 54.5 ± 1.8 kg, height 1.63 ± 0.73 m and body fat 19.4 ± 1.3%). Non statistical significance was found between the characteristics of the study participants. Hydration assessment showed that based on the USG criteria (<1.020), 96.7% (30 out of 31) and 91.7% (56 out of 61) of the CON and INT group, respectively, were dehydrated on the second day of the camp. At the fourth day, 96.7% (30 out of 31) and 66.1% (40 out of 61) of the CON and INT group, respectively, were classified as dehydrated. The data showed that a significantly smaller percent of subjects was classified as dehydrated as a response of the intervention (*P*=0.003) (Fig. 1).

**Fig. 2:**
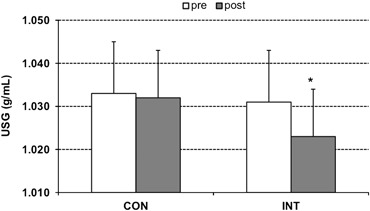
Urine osmolality before and after the intervention in the control and intervention group. ^*^Statistically significant difference between pre and post in each group.

As shown in Fig. 2, when dehydration was assessed based on the urine osmolality (>700 mOsm/kg water), then 90.3% (28 out of 31) and 83.3% (51 out of 61) of the CON and INT group, respectively, were dehydrated before the intervention (second day). As a response to the intervention, the percentage of the subjects that were classified as dehydrated in the INT group decreased to 62.1% (*P*=0.005), while there was no difference in the CON group (90.0%).

Table 2 presents the results in the performance tests performed by the children. Endurance performance in the 600 m running test was improved only in INT [pre: 189 ± 5 s, post: 167 ± 4 s (*P*=0.009)] while no differences were found in the heart rate immediately after the test [pre: 191 ± 1 bpm, post: 190 ± 1 bpm (*P*>0.05)]. No differences were found between the pre and post intervention in both of the groups in sprint performance and vertical jump. Skill test score for the CON group was 1.48 ± 1.0 and 1.48 ± 1.17, pre and post, respectively (*P*>0.05). For the INT group, the same score was 1.50 ± 1.0 and 1.55 ± 1.0, pre and post, respectively (*P*>0.05).

**Table 2:** Performance tests

Test	CON_pre_	CON_post_	% change	INT_pre_	INT_post_	% change
600 m (s)	186 ± 10	177 ± 5	−7.7 ± 2.2	189 ± 5	167 ± 4*	−12.7 ± 1.5
30 m (s)	5.6 ± 0.1	5.6 ± 0.1	0.74 ± 1.06	5.7 ± 0.5	5.6 ± 0.6	−1.07 ± 0.72
Vertical jump (cm)	26.3 ± 1.5	25.3 ± 1.8	−0.9 ± 1.05	25.0 ± 1.0	24.6 ± 1.1	−0.4 ± 1.10
Skill test	1.5 ± 1.0	1.5 ± 1.2	0.4 ± 0.9	1.5 ± 1.0	1.6 ± 1.0	0.9 ± 0.3

Values are means ± SE.

* Statistically significant difference between pre and post.

At the end of the camp, athletes were asked different questions related to self-perception of the hydration. The results presented in Table 3, were reported to a score ranging from 1 to 10 (1=very bad, 5=ok, 10=great job) for the CON and INT group. From the environmental symptoms questionnaire, no differences were found among the groups in symptoms related to heat or dehydration.

**Table 3:** Hydration awareness questionnaire scores

Questions	CON	INT
Do you think you did a good job keeping yourself hydrated during the camp?	8.2 ± 2.4	9.0 ± 1.9
Did the camp encourage hydration?	7.2 ± 3.0	9.3 ± 1.6
Did the camp allow you time to drink?	8.4 ± 2.4	9.9 ± 1.9
Were fluids readily accessible at the camp?	9.4 ± 1.7	9.5 ± 1.9
Was there anything that prevented you from hydrating during the camp?	0.2 ± 0.4	0.04 ± 0.2

Values are means ± SE.

## Discussion

The main finding of the study was that a relative simple but comprehensive intervention program proved successful for enhancing hydration status over just a 2-day period. Additionally, the improvement of hydration status through ad libitum water intake, in free-living young athletes training in the heat, led to significant increases in endurance exercise performance.

To our knowledge, this research is the first field study which finds enhanced performance related to hydration status in the rarely studied youth population. A possible explanation for this improvement in endurance performance time, taking into consideration the fact that no differences were found in the heart rate immediately after the 600 m performance test, is that stroke volume was better preserved, consequently cardiac output was maintained and as a final result, aerobic capacity was significantly improved. These conclusions are in accordance with the results by Gonzalez-Alonso et al. (2000). Interestingly in a recent study, significantly greater heart rate was observed in dehydrated athletes at the end of submaximal or 10 and 20 min after the maximal trail running (Casa et al., 2010). However, in the present study the duration of the performance test was shorter and more intense inducing in both trials the same, near maximal, heart rate. Similarly with Casa et al. (2010), no differences in heart rate during exercise were found during maximal exercise.

Furthermore, although the improvement observed in performance time could be influenced by a learning effect from the first trial, no performance enhancement was revealed in the control group, supporting the finding of this investigation.

The high incidence of dehydration observed during the summer camp, pointed out by the USG and Uosm values on the baseline measurements, is in accordance with the values measured by two indicative studies, which found consistently high baseline USG values of young soccer players, resulting in the fact that athletes showed up to practice in a hypohydrated state (Stover et al., 2006; Kotsis et al., 2007). These high values indicate that for the exercising children, education concerning the deleterious effects of dehydration and the possibility of heat injuries during exercise and on the other hand, the beneficial effects of optimal hydration status should be a priority at camps, games and practices.

However, the intervention program that was used through the lectures concerning the importance of good hydration on health and the free access to fluids resulted in a significant decline in the percentage of the dehydrated children in the intervention group. In a recent study, it was concluded that the provision of two 591 mL bottles of water or sport drink, one consumed between dinner and sleep and the other before morning training, is a simple method which leads to significant improvement to the hydration status of high-school football players, as indicated by USG values (Stover et al., 2006). Therefore, intervention to teach and facilitation of hydration accessibility, along with simple and realistic hydration strategies will benefit youths exercising at summer camps.

There is a plethora of investigations examining fluid intake before, during and after training and competition that have suggested that a lack of adequate fluid intake impairs or decreases physical performance (Sawka et al., 1983; Cadarette et al.,1984; Montain & Coyle, 1992; Gonzalez-Alonso et al., 1995, 1997; Armstrong et al., 1997; Maughan, 2003). To our knowledge, no study until now has examined the effect of hydration status on endurance performance in children. From the data presented, it is documented that free accessibility in water and education concerning the benefits of optimal hydration status, which in turn increased the consumption of fluids, led to a significant improvement to the endurance test (600 m) performed by children.

Nevertheless, no significant differences were observed between the two groups in sprint performance, vertical jump and skill tests. In a review by Maughan (2003), it is shown that muscle strength and sprint performance appear to remain relatively unaffected under hypohydration state, explaining in part the observed non-significant results in sprint performance and vertical jump. However, Shirreffs et al. (2004) suggests that mild dehydration reduces the subjective perception of alertness and ability to concentrate.

Finally, in the present study it was observed that the subjects in both the control and the intervention group were in a hypohydrated state throughout their stay in the summer camp. Even though, most of the athletes in the control group and in the intervention group remained dehydrated they perceived that were doing good job staying hydrated (8.2 out of 10). In accordance with the results by a recent study, the children in the camp generally recognized whether they were doing a good or a bad job hydrating (Decher et al., 2008). However, they established a lack of transforming the hydration knowledge into effective hydration strategies. At last, it is important to mention the lack of any significant differences through the environmental symptoms questionnaire, which was provided, probably due to the fact that it depicts incidence and severity of symptoms produced by exposure to extreme climatic conditions (Sampson & Kobrick, 1980).

## Perspectives

Few studies have investigated the capacity of children exercising in the heat and the relationship of their hydration status and performance. In the present study, we demonstrated that the improvement in hydration status, through an educational intervention led to significant enhancement in endurance performance in exercising children. It is obvious that continued efforts must be made by coaches, camp staff and athletes, in order to educate the youths toward the benefits of optimal hydration state, the development of more efficient hydration strategies and the methods of assessing and monitoring their hydration status.
